# Enhanced protective immunity of the chimeric vector-based vaccine rAdV-SFV-E2 against classical swine fever in pigs by a Salmonella bacterial ghost adjuvant

**DOI:** 10.1186/s13567-016-0346-9

**Published:** 2016-06-14

**Authors:** Shui-Li Xia, Jian-Lin Lei, Mingliang Du, Yimin Wang, Xin Cong, Guang-Tao Xiang, Lian-Feng Li, Shenye Yu, Enqi Du, Siguo Liu, Yuan Sun, Hua-Ji Qiu

**Affiliations:** State Key Laboratory of Veterinary Biotechnology, Harbin Veterinary Research Institute, Chinese Academy of Agricultural Sciences, Harbin, 150001 China; College of Veterinary Medicine, Northwest A&F University, Yangling, 712100 Shaanxi China

## Abstract

Classical swine fever (CSF) is a highly contagious swine disease caused by classical swine fever virus (CSFV). Previously, we demonstrated that rAdV-SFV-E2, an adenovirus-delivered, Semliki Forest virus replicon-vectored marker vaccine against CSF, is able to protect pigs against lethal CSFV challenge. From an economical point of view, it will be beneficial to reduce the minimum effective dose of the vaccine. This study was designed to test the adjuvant effects of *Salmonella enteritidis*-derived bacterial ghosts (BG) to enhance the protective immunity of rAdV-SFV-E2 in pigs. Groups of 5-week-old pigs (*n* = 4) were immunized intramuscularly twice with 10^5^ median tissue culture infective doses (TCID_50_) rAdV-SFV-E2 combined with 10^10^ colony forming units (CFU) BG, 10^6^ or 10^5^ TCID_50_ rAdV-SFV-E2 alone or 10^10^ CFU BG alone at an interval of 3 weeks, and challenged with the highly virulent CSFV Shimen strain at 1 week post-booster immunization. The results show that the pigs inoculated with 10^5^ TCID_50_ rAdV-SFV-E2 plus BG or 10^6^ TCID_50_ rAdV-SFV-E2 alone were completely protected from lethal CSFV challenge, in contrast with the pigs vaccinated with 10^5^ TCID_50_ rAdV-SFV-E2 or BG alone, which displayed partial or no protection following virulent challenge. The data indicate that BG are a promising adjuvant to enhance the efficacy of rAdV-SFV-E2 and possibly other vaccines.

## Introduction

Classical swine fever (CSF) is one of the most contagious diseases and characterized by high fever and high mortality, resulting in huge economic losses to the pig industry [1]. Classical swine fever virus (CSFV), one of the members of the *Pestivirus* genus in the *Flaviviridae* family, is the causative pathogen of CSF. The genome of CSFV is a single-stranded, positive-sense RNA of about 12.3 kb, which encodes a polyprotein that is processed co- and posttranslationally into 12 proteins (N^pro^-C-E^rns^-E1-E2-p7-NS2-NS3-NS4A-NS4B-NS5A-NS5B) [2, 3].

Currently, immunization with modified live vaccines (MLV, e.g. C-strain) is a major strategy to control CSF in many countries [4]. However, the European Union has banned vaccination using traditional CSF MLV against CSF since 1990, as antibodies induced by MLV or field CSFV strains cannot be distinguished serologically [5]. Therefore, developing a safe and effective marker vaccine allowing differentiation of infected from vaccinated animals (DIVA) is very important. To address this issue, we developed a marker CSF vaccine rAdV-SFV-E2 based on human adenovirus type 5 (HAdV-5)/alphavirus replicon chimeric vector. We demonstrate that rAdV-SFV-E2 can elicit strong cellular and humoral responses in pigs and provide sterile immunity and complete protection against lethal CSFV challenge comparable to the C-strain [6, 7]. From an economic point of view, it is necessary to reduce the minimum effective dose (MED) of the vaccine.

Co-administration of adjuvants, such as aluminum and mineral oil, is an effective method to improve the efficacy of a suboptimal vaccine. Adjuvants can help antigens in activating pathways significantly in the induction of innate immunity, predominantly targeting antigen-presenting cells (APC) and consequently influencing the adaptive immune response [8]. Well-characterized bacterial ghosts (BG)-based adjuvants have unique advantages. BG are nonliving cell envelope preparations from Gram-negative bacteria, devoid of cytoplasmic contents, while their cellular morphology and native surface antigenic structures remain preserved. So they are potentially powerful adjuvants due to the presence of bacterial membrane components such as lipopolysaccharides, peptidoglycans and monophosphoryl lipid A (MPL) [9]. MPL interacts with toll-like receptor 4 [10], induces the production and release of cytokines [11] and increases the migration and maturation of dendritic cells [12]. Owing to the particulate nature of BG and the fact that they contain many well-known immune-stimulating compounds, BG have the potential to enhance immune responses to various antigens [13]. Therefore, we hypothesize that rAdV-SFV-E2 with BG can provide a better protection against CSF in pigs.

The present study was aimed at evaluating the adjuvant effects of BG to enhance the protective immunity of rAdV-SFV-E2 in pigs.

## Materials and methods

### Bacterial ghost adjuvant, vaccines and viruses

The *Salmonella enteritidis*-derived BG adjuvant was produced by controlled expression of the modified lysis gene E (mE) from bacteriophage ΦX174 [14]. Briefly, *S. enteritidis* DH091 harboring the recombinant bacteriolytic plasmid pBV-mE expressing the mE that is able to lyse the bacteria when induced at 42 °C, was cultured to an OD_600nm_ of 1.0 at 37 °C. Then the culturing temperature was raised to 42 °C for mE expression, resulting in lysis of the bacteria. After 1 h, when the lysis curve started to decline, 10 μL of the cell suspension was spread onto LB plates containing ampicillin, followed by a 12-h incubation at 37 °C. Viable colonies were determined as colony forming units (CFU)/mL. The OD_600nm_ was measured every 15 min till no further decline in OD_600nm_. After lysis, the BG were harvested by centrifugation (4000 × *g* for 10 min), washed with PBS (pH 7.2), suspended in 20 mL of sterile distilled water, lyophilized and stored at −20 °C. rAdV-SFV-E2 is an adenovirus-delivered, alphavirus replicon-vectored vaccine encoding the E2 glycoprotein of CSFV [6]. The highly virulent CSFV Shimen strain [7] maintained at Harbin Veterinary Research Institute (HVRI) was used for challenge.

### Animals

Twenty 5-week-old cross-bred weanling piglets, free of CSFV-specific antibodies and antigens, were raised in the animal facility at HVRI. All experimental procedures involving animals were approved by the Experimental Animal Ethics Committee of HVRI.

### Immunization-challenge experiment

The piglets were randomly divided into five groups of four animals each. Groups A and C were respectively vaccinated with 10^6^ TCID_50_ and 10^5^ TCID_50_ rAdV-SFV-E2 alone; Group B were co-immunized intramuscularly with 10^5^ TCID_50_ rAdV-SFV-E2 and 10^10^ CFU BG; Groups D and E were injected intramuscularly with 10^10^ CFU BG and DMEM (2 mL), respectively, serving as controls. Three weeks later, all the pigs were given a booster immunization with the same vaccine, dose and route of administration. All the pigs were challenged intramuscularly with 10^6^ TCID_50_ CSFV Shimen strain 1 week post-booster immunization. Following challenge, the rectal temperature and clinical signs were recorded every day. All the pigs were euthanized at 15 days post-challenge (dpc). The tissues from all the pigs were subjected to pathological examinations as described previously [15].

### Serological assays

Serum samples were collected at different time points post-immunization. The presence of the E2-specific antibodies in samples were tested using the IDEXX HerdChek* CSFV antibody test kit (IDEXX Laboratories, Shiphol-Rijk, The Netherlands).

To test the level of CSFV-specific neutralizing antibodies (NAbs), a serum-virus neutralization test (SVNT) was carried out in 96-well flat-bottom microtiter plates (Coring, USA) as described previously [16].

### Real-time RT-PCR

The total RNA was extracted from EDTA-treated blood samples collected at different days after challenge, and detection of CSFV RNA was performed by real-time RT-PCR with a CSFV-specific probe (5′-FAM-AGG ACT AGC AAA CGG AGG GAC TAG CCG-TAMRA-3′) and a primer pair (5′-GAA CTG GGC TAG CCA TG-3′ and 5′-ACT GTC CTG TAC TCA GGA C-3′) [17].

### Pathology

All surviving pigs were euthanized at 15 dpc. Various organs (spleen, kidney, tonsils, lymph nodes and bladder) were collected and subjected to pathological and histopathological examinations as described previously [6, 18].

### Statistical analysis

Statistical analysis was conducted using the SPSS 14.0 software. One-way ANOVA followed by Duncan’s multiple-range tests were used to compare the parameters among the different groups.

## Results

### Antibody production

**Figure 1:**
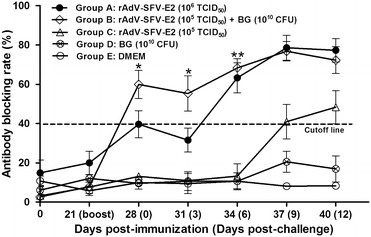
**Detection of serum antibodies in immunized pigs by blocking ELISA.** Five groups of pigs (*n* = 4) were immunized and challenged as described in the “Materials and methods” section. Serum samples were taken at 21 days post-immunization and 0 (7 days post-booster immunization), 3, 6, 9 and 12 days post-challenge and tested for CSFV-specific NAbs. Standard deviations were shown as error bars. *Significant difference between Groups B and C (*P* < 0.05); **very significant difference between Groups B and C (*P* < 0.001); CFU: colony forming units.

E2-specific antibodies and NAbs were tested by blocking ELISA and SVNT following vaccination and challenge. The E2-specific antibodies were first detected in all the pigs in Group B (10^5^ TCID_50_ rAdV-SFV-E2 plus 10^10^ CFU BG) at 1 week post-booster immunization, with the mean antibody blocking rate of 59.91%. Most pigs (3/4) in Group A (10^6^ TCID_50_ rAdV-SFV-E2 alone) seroconverted at 1 week post-booster immunization. After challenge, the anti-E2 antibodies in Groups A and B decreased transiently at 0–3 dpc and then increased sharply after 3 dpc, and the antibody titers peaked at 9 dpc, with the mean antibody blocking rates of about 80%. The E2-specific antibodies were undetectable in Group C (10^5^ TCID_50_ rAdV-SFV-E2 alone) prior to challenge and detected at 9 dpc, with the mean antibody blocking rates of 40.96%. As expected, no E2-specific antibody was detected in Groups D (10^10^ CFU BG alone) and E (DMEM) throughout the experiment. There was a significant difference at 0 and 3 dpc (*P* < 0.05), a very significant difference at 6 dpc (*P* < 0.001) between Groups B and C, and no significant difference in antibody titers between Groups A and B during the experiment (*P* > 0.05) (Figure 1).

Based on SVNT, anti-CSFV NAbs were also tested. At 21 days post-vaccination (dpv), anti-CSFV NAbs were detected in Group B and the mean neutralization titers were 1:14. The neutralization titers rose to a peak at 28 dpv (7 days post-booster immunization), with 1:122 in Group A, 1:164 in Group B. Following virulent challenge, anti-CSFV NAbs levels increased remarkably after a transient slight decrease in Group A or B, and at 9 dpc, with mean neutralization titers of 1:841 for Group A, 1:612 for Group B. In Group C, anti-CSFV NAbs were detected at 9 dpc, with mean neutralization titers of 1:194. No neutralizing titers were detectable in Groups D and E. There was a significant difference in anti-CSFV NAbs levels between Groups B and C at 9 dpc (*P* < 0.05) (Table 1).

**Table 1:** **CSFV-specific neutralizing antibodies in pigs following lethal CSFV challenge**

Groups	Days post-immunization (days post-challenge)					
	0	21	28 (0)	31 (3)	34 (6)	37 (9)
A: rAdV-SFV-E2 (10^6^ TCID_50_)	<10	<10	122 ± 52	62 ± 9	699 ± 88	841 ± 258
B: rAdV-SFV-E2 (10^5^ TCID_50_) + BG (10^10^ CFU)	<10	14 ± 2	164 ± 39	147 ± 65	484 ± 66	612 ± 89*
C: rAdV-SFV-E2 (10^5^ TCID_50_)	<10	<10	<10	<10	<10	194 ± 83
D: BG (10^10^ CFU)	<10	<10	<10	<10	<10	<10
E: DMEM	<10	<10	<10	<10	<10	<10

Five groups of pigs (*n* = 4) were immunized and challenged as described in the “Materials and methods” section. Serum samples were collected at different times following immunization and challenge and subjected to detection of anti-CSFV neutralizing antibody titers by serum–virus neutralization test. The diluted sera were mixed with equal volume of 200 TCID50 CSFV Shimen strain and incubated for 60 min at 37 °C. The serum–virus mixtures were inoculated to confluent PK-15 cells cultured in 96-well plates and incubated for 60 min at 37 °C. The inoculated cells were then incubated for 72 h at 37 °C. IFA was performed as described previously [6]. The cells were examined under a fluorescence microscope, and the titers of CSFV-specific neutralizing antibodies were determined and expressed as the reciprocal of the highest dilution at which infection of the PK-15 cells was inhibited in 50% of the culture wells.

* Significant difference between groups B and C (*P* < 0.05).

CFU: colony forming units.

### Clinical protection of vaccinated pigs from virulent CSFV challenge

No adverse reactions were observed in any pigs following immunization. Following virulent challenge, no clinical symptoms were observed in Group A or B. Three out of four pigs in Group C exhibited a short-term fever (ranging from 40.5 to 42 °C), and all returned to normal 2–5 days later and survived at 15 dpc. One pig in Group C and all the pigs in Groups D and E showed typical CSF clinical signs, such as fever, inappetence, depression, chill, constipation, prostration and incoordination, followed by diarrhea, locomotorataxia and posterior paresis from 3 dpc to the end of the experiment. The fever frequencies in Groups D (27/46) and E (27/44) were the highest, followed by Group C (14/50) (Table 2).

**Table 2:** **Clinical outcome of the immunized pigs following virulent challenge**

Groups	Days to fever onset	Fever rate^a^	Fever frequency^b^	Survival rate
A: rAdV-SFV-E2 (10^6^ TCID_50_)	–	0/4	0/60	4/4
B: rAdV-SFV-E2 (10^5^ TCID_50_) + BG (10^10^ CFU)	–	0/4	0/60	4/4
C: rAdV-SFV-E2 (10^5^ TCID_50_)	3	3/4	14/50	3/4
D: BG (10^10^ CFU)	3	4/4	27/46	0/4
E: DMEM	3	4/4	27/44	0/4

Five groups of pigs (*n* = 4) were immunized and challenged as described in the “Materials and methods” section. Following challenge, the clinical signs and rectal temperatures were recorded daily. Fever is defined as rectal temperature ≥40.5 °C.

*–* no fever, CFU: colony forming units.

^a^Numbers of pigs showing fever/total numbers of pigs in each group.

^b^Total days with any pig showing fever/total days of monitored for all the pigs in a group following virulent challenge.

### Virological protection of vaccinated pigs from virulent CSFV challenge

Viral RNA was undetectable in Group A or B. Low-level viral RNA (about 10^3^ copies/μL) was detected in some pigs (2/4) in Group C at 6, 9 and 12 dpc. In Groups D and E, viral RNA loads higher than 10^3^ copies/μL were detected from 6 dpc to death (Table 3).

**Table 3:** **Detection of viral RNA in whole blood samples from immunized pigs after virulent challenge by real-time RT-PCR**

Groups	Pig No.	Days post-challenge				
		0	3	6	9	12
A: rAdV-SFV-E2 (10^6^ TCID_50_)	A1	–	–	–	–	–
	A2	–	–	–	–	–
	A3	–	–	–	–	–
	A4	–	–	–	–	–
B: rAdV-SFV-E2 (10^5^ TCID_50_) + BG (10^10^ CFU)	B1	–	–	–	–	–
	B2	–	–	–	–	–
	B3	–	–	–	–	–
	B4	–	–	–	–	–
C: rAdV-SFV-E2 (10^5^ TCID_50_)	C1	–	–	–	–	–
	C2	–	–	1.49 × 10^4^	3.70 × 10^3^	/
	C3	–	–	–	–	–
	C4	–	–	2.70 × 10^3^	4.40 × 10^3^	1.97 × 10^2^
D: BG (10^10^ CFU)	D1	–	–	2.35 × 10^4^	5.04 × 10^4^	/
	D2	–	–	4.86 × 10^4^	2.43 × 10^4^	2.55 × 10^5^
	D3	–	–	5.04 × 10^5^	6.96 × 10^5^	/
	D4	–	–	3.44 × 10^4^	6.81 × 10^3^	1.68 × 10^4^
E: DMEM	E1	–	–	1.31 × 10^4^	2.67 × 10^4^	/
	E2	–	–	4.21 × 10^5^	4.70 × 10^4^	9.08 × 10^4^
	E3	–	–	9.76 × 10^4^	6.55 × 10^5^	/
	E4	–	–	3.06 × 10^5^	3.92 × 10^3^	/

Five groups of pigs (*n* = 4) were immunized and challenged as described in the “Materials and methods”. Whole blood samples were collected at days 0, 3, 6, 9 and 12 post-challenge. CSFV RNA was extracted and quantified by a real-time RT-PCR described previously [17].

*–* not detectable, / died, CFU: colony forming units.

### Pathological protection of vaccinated pigs from lethal CSFV challenge

**Figure 2:**
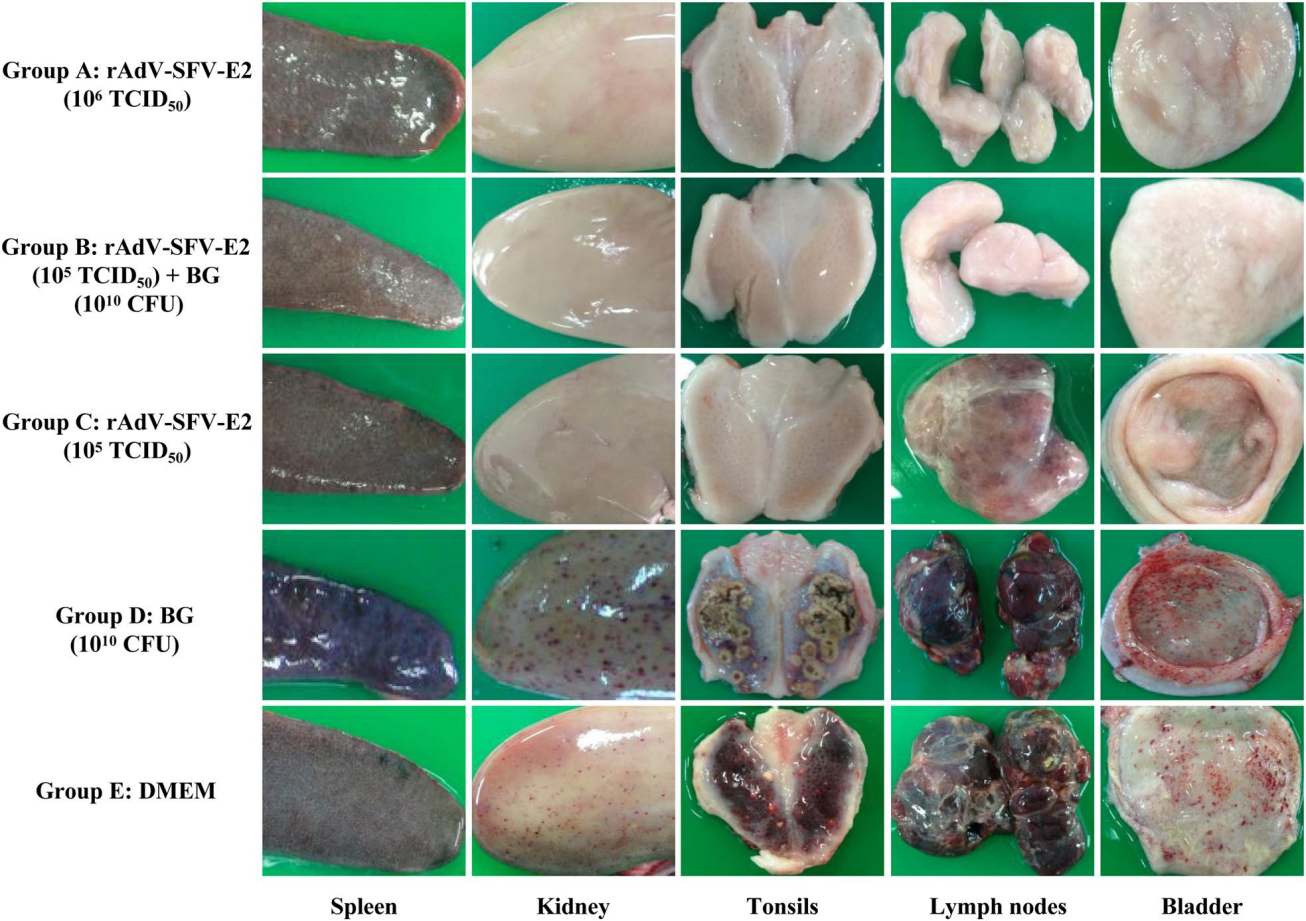
**Representative pathological changes of immunized pigs challenged with CSFV Shimen strain.** Five groups of pigs (*n* = 4) were immunized and challenged as described in the “Materials and methods” section. At 15 days post-challenge (dpc), all surviving pigs were euthanized and different tissues (spleen, kidney, tonsils, lymph nodes and bladder) were collected and pathological examinations performed. CFU: colony forming units.

At 15 dpc, all surviving pigs were euthanized and subjected to pathological and histopathological examinations. All the pigs in Groups A and B did not show any pathological changes. Most pigs (3/4) in Group C showed mild lesions (including infarcts in the spleen, slight hemorrhages in the lymph nodes and necrotic foci in the tonsils). Similar severe pathological changes were observed in one pig of Group C and all the pigs of Groups D and E, including infarcts in the spleen, massive petechiae in the kidney and bladder, hemorrhages with necrotic foci in the tonsils, enlargement and hemorrhage of the lymph nodes and button-like ulcers in the ileocecal valve (Figure 2).

**Figure 3:**
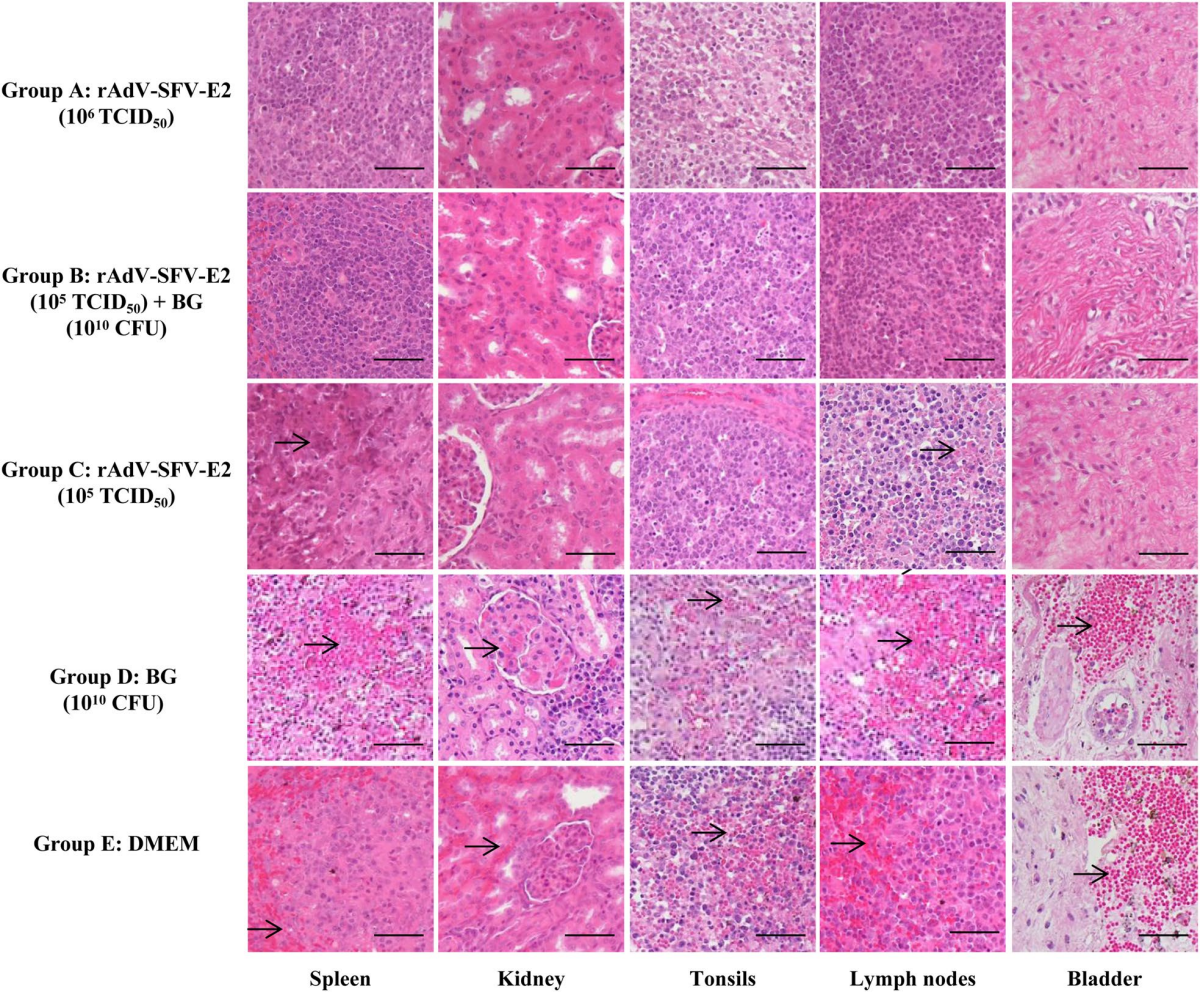
**Representative histopathological changes in pigs challenged with CSFV Shimen strain.** Five groups of pigs (*n* = 4) were immunized and challenged as described in the “Materials and methods” section. At 15 days post-challenge (dpc), various tissues (spleen, kidney, tonsils, lymph nodes and bladder) were collected from the challenged animals, fixed with buffered 4% formalin and subsequently embedded in paraffin wax. Tissue sections (around 4-μm thick) were prepared and stained with hematoxylin and eosin for histopathological examinations. In Groups D and E, severe hemorrhages in the lymph nodes, tonsils, spleen, kidney and bladder were indicated as arrows; in Group C, slight to moderate histopathological changes were found in some tissues, such as focal necrosis in the splenic parenchyma (arrow) and depletion of lymphocytes (arrow) in the white pulp in the spleen and hemorrhages (arrow) in the lymph nodes. All the pigs in Group A or B did not show any histopathological changes. CFU: colony forming units. Bars 50 μm.

No histopathological changes were observed for pigs in Groups A and B. The pigs in Group C displayed slight to moderate histopathological changes in some tissues, including focal necrosis in the splenic parenchyma and depletion of lymphocytes in the white pulp in the spleen and hemorrhages in the lymph nodes. The pigs in Groups D and E displayed similar severe histopathological changes in most tissues, including depleted lymphoid follicles and hemorrhages in the lymph nodes and tonsils, diffuse hemorrhages throughout the splenic parenchyma and depletion of lymphocytes in the white pulp in the spleen, hemorrhages in the interstitial spaces in the kidney and diffuse hemorrhages in the bladder (Figure 3).

## Discussion

In this study, the efficacy of rAdV-SFV-E2 in combination with BG was evaluated. The results show that pigs injected with 10^5^ TCID_50_ rAdV-SFV-E2 plus 10^10^ CFU BG provided complete protection against lethal CSFV challenge and the efficacy was comparable to 10^6^ TCID_50_ rAdV-SFV-E2, which indicates that BG can decrease the effective immunization dose of rAdV-SFV-E2 by at least 10-fold.

Adjuvants, such as microbial proteins or carbohydrates may activate APC and induce specific immune responses [19]. BG have the same potential because of their adjuvant components [20, 21]. A recent study has demonstrated that internalization of BG by porcine APC leads to enhanced expression of antigen-presenting molecules on the surface of APC and significantly increases the antigen-presenting capacity of APC [20]. Therefore, more memory B cells were generated following immunization with rAdV-SFV-E2 plus BG than rAdV-SFV-E2 alone, since more antigens were presented to B cells by activated APC. Another possible mechanism might be the effective delivery of rAdV-SFV-E2 by BG, since BG can function as carriers of protein antigens, drugs and a high loading capacity for DNA [9, 21]. Thus, immunization with BG-adjuvanted rAdV-SFV-E2 induced higher E2-specific antibodies and NAbs than rAdV-SFV-E2 alone after booster immunization (Figure 1; Table 1).

No significant difference in serum interferon γ (IFN-γ) and interleukin 4 (IL-4) was found between Groups B (10^5^ TCID_50_ rAdV-SFV-E2 plus 10^10^ CFU BG) and C (10^5^ TCID_50_ rAdV-SFV-E2) (data not shown). Therefore, it is necessary to evaluate cellular immune responses in details in future work, especially the CSFV-specific CD8^+^ cytotoxic T lymphocytes, which represent an important defense mechanism in the elimination of cells infected by CSFV [5, 22, 23]. In this study, we also found that some pigs immunized with BG displayed transient allergic reactions. In the following experiments, different amounts of the BG will be evaluated and compared with several commonly used adjuvants regarding the enhancement of the protective immunity of rAdV-SFV-E2.

In conclusion, the BG adjuvant can significantly enhance the protective immunity induced by the chimeric vector-based vaccine rAdV-SFV-E2 against CSF in pigs and it may be a promising adjuvant for other vaccines.
